# Supplementary material for: Adolescent Pregnancy in South Asia: A Systematic Review of Observational Studies
Source: Int J Environ Res Public Health. 2022 Nov 15;19(22):15004. doi: 10.3390/ijerph192215004 (PMC9690629; doi:10.3390/ijerph192215004)
Supplement: Supplementary file 1 [file ijerph-19-15004-s001.zip › Supplementary Table S1.pdf]

Supplementary Table S1: Quality assessment of selected cross-sectional and cohort studies

Author [Ref.] Year country	Was the research question or objective in this paper clearly stated?	Was the study population clearly specified and defined?	Was the participation rate of eligible persons at least 50%?	Were all the selected or recruited from the same or similar populations (including the same time period)? Were inclusion and exclusion criteria for being in the study prespecified and applied uniformly to all participants?	Was a sample size justification, power description, or variance and effect estimates provided?	For the analyses in this paper, were the exposure(s) of interest measured prior to the outcome(s) being measured?	Was the timeframe sufficient so that one could reasonably expect to see an association between exposure and outcome if it existed?	For exposures that can vary in amount or level, did the study examine different levels of the exposure as related to the outcome (e.g., categories of exposure, or exposure measured as continuous variable)?	Were the exposure measures (independent variables) clearly defined, valid, reliable, and implemented consistently across all study participants?	Was the exposure(s) assessed more than once over time?	Were the outcome measures (dependent variables) clearly defined, valid, reliable, and implemented consistently across all study participants?	Were the outcome assessors blinded to the exposure status of participants?	Was loss to follow-up after baseline 20% or less?	Were key potential confounding variables measured and adjusted statistically for their impact on the relationship between exposure(s) and outcome(s)?
Ali et al., 2021, Bangladesh	YES	YES	NR	YES	YES	No	No	YES	YES	NO	YES	NA	NA	NA
Sarder et al., 2020 Bangladesh	YES	YES	NR	YES	YES	No	No	YES	YES	NO	YES	NA	NA	NA
Alam et al., 2018, Bangladesh	YES	YES	YES	YES	NO	No	No	YES	YES	NO	YES	NA	NA	NA
Haq et al., 2018, Bangladesh	YES	YES	NR	YES	NO	No	No	YES	YES	NO	YES	NA	NA	NA
Islam et al., 2017, Bangladesh	YES	YES	NR	YES	NO	No	No	YES	YES	YES	YES	NA	NA	NA
Sayem et al., 2011, Bangladesh	YES	YES	YES	YES	NO	No	No	NA	YES	NO	YES	NA	NA	NA
Gurung et al., 2020, Nepal	YES	YES	NO	NR	NO	No	No	NA	YES	NO	NO	NA	NA	NA
Poudel et al., 2018, Nepal	YES	YES	NR	YES	NO	No	No	YES	YES	YES	YES	NA	NA	NA
Devkota et al., 2018, Nepal	YES	YES	NR	YES	NO	No	No	NO	YES	NO	YES	NA	NA	YES
Pradhan et al., 2018, Nepal	YES	YES	NR	YES	NO	No	No	YES	YES	YES	YES	NA	NA	NA
Ali et al., 2021, Pakistan	YES	YES	NR	YES	NO	No	No	YES	YES	YES	YES	NA	NA	NA
Agampodi et al., 2021, Sri Lanka	YES	YES	YES	NO	NO	Yes	YES	YES	YES	YES	YES	NR	NR	YES

Note: YES=1, NO=0, Not Applicable (NA)=0, and Not Reported (NR)=0.
