# Supplementary material for: Adolescent Pregnancy in South Asia: A Systematic Review of Observational Studies
Source: Int J Environ Res Public Health. 2022 Nov 15;19(22):15004. doi: 10.3390/ijerph192215004 (PMC9690629; doi:10.3390/ijerph192215004)
Supplement: Supplementary file 1 [file ijerph-19-15004-s001.zip › Supplementary Table S2.pdf]

Supplementary Table S2: Quality assessment of selected case-control studies

Author [Ref.] Year Country	Was the research question or objective in this paper clearly stated?	Was the study population clearly specified and defined?	Did the authors include a sample size justification?	Did controls selected or recruited from the same or similar population that gave rise to the cases (including the same timeframe)	Were the definitions, inclusion and exclusion criteria, algorithms or processes used to identify or select cases and controls valid, reliable, and implemented consistently across all study participants?	Were the cases clearly defined and differentiated from controls?	If less than 100 percent of eligible cases and/or controls were selected for the study, were the cases and/or controls randomly selected from those eligible?	Was the use of concurrent controls?	Were the investigators able to confirm that the exposure/risk occurred prior to the development of the condition or event that defined a participant as a case?	Were the measures of exposure/risk clearly defined, valid, reliable, and implemented consistently (including the same time) across all study participants?	Were the assessors of exposure/risk blinded to the case or control status of participants?	Were key potential confounding variables measured and adjusted statistically in the analyses? If matching was used, did the investigators account for matching during study analysis?
Neupane et al., 2019, Nepal	YES	YES	NO	NO	YES	YES	YES	NO	NO	YES	NR	NO
Sharma et al., 2002, Nepal	YES	YES	NO	YES	YES	YES	YES	NO	NO	YES	NR	NO
Dulitha et al., 2013, Sri Lanka	YES	YES	NO	NO	YES	YES	YES	NO	NO	YES	NR	NO

Note: YES=1, NO=0, Not Applicable (NA)=0, and Not Reported (NR)=0.
